# Definitions matter: Multicenter investigation of incidence and outcome of poor graft function after hematopoietic cell transplantation

**DOI:** 10.1002/hem3.70059

**Published:** 2024-12-17

**Authors:** Konradin F. Müskens, Winny N. R. Collot‐d'Escury, Rana Dandis, Saskia Haitjema, Jürgen Kuball, Moniek A. de Witte, Marc Bierings, Caroline A. Lindemans, Stefan Nierkens, Mirjam E. Belderbos

**Affiliations:** ^1^ Princess Máxima Center for Pediatric Oncology Utrecht The Netherlands; ^2^ Central Diagnostic Laboratory, University Medical Center Utrecht Utrecht University Utrecht The Netherlands; ^3^ Department of Hematology, University Medical Center Utrecht Utrecht University Utrecht The Netherlands; ^4^ Laboratory of Translational Immunology University Medical Center Utrecht Utrecht University Utrecht The Netherlands; ^5^ Wilhelmina Children's Hospital, University Medical Center Utrecht Utrecht University Utrecht The Netherlands

## Abstract

Despite advances in allogeneic hematopoietic cell transplantation (HCT), poor graft function (PGF) remains an important complication with substantial morbidity and mortality. The investigation of preventive and therapeutic PGF treatments is hindered by inconsistencies in reported incidence and outcomes across studies, which may be explained by heterogeneity in PGF definition. To assess the impact of definition heterogeneity, we conducted a multicenter study, analyzing over 35.000 longitudinal blood counts from 427 pediatric and 405 adult HCT recipients. We compared the incidence, risk factors, and outcome of PGF, based on the three most common definitions. We identified 97 pediatric and 75 adult HCT recipients fulfilling at least one PGF definition. The 2‐year cumulative incidence of PGF varied significantly depending on the definition used, ranging from 6.8% to 20% in children and 4.9% to 18% in adults. Two‐year mortality for PGF patients ranged from 33% to 40% in children and 46% to 65% in adults. Notably, PGF patients identified solely by lenient definitions had similar mortality to HCT recipients with good graft function. Risk factors for PGF also varied by definition in both cohorts, and included older recipient age and cord blood transplantation. In conclusion, our study demonstrates that differences in PGF definition significantly impact the reported incidence, risk factors, and outcome. This underscores the need to harmonize PGF definitions across scientific studies, clinical practice, and transplant registries. Future studies, using standardized, quantitative thresholds for PGF, are required to determine optimal treatment strategies for both mild and severe forms of PGF.

## Graphical abstract

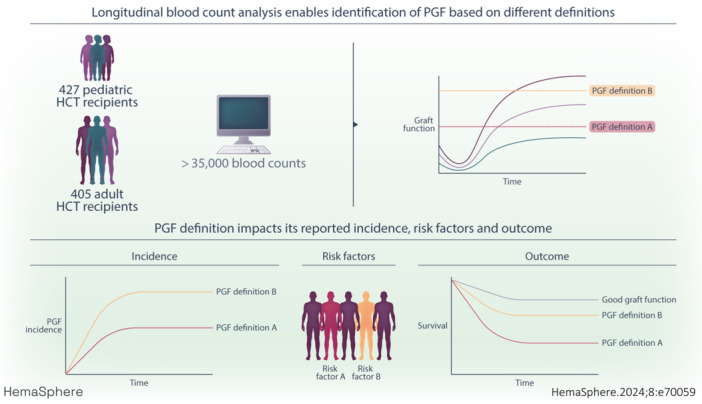

## INTRODUCTION

Allogeneic hematopoietic cell transplantation (HCT) is a life‐saving therapy for a wide range of diseases, including hematologic malignancies, bone marrow failure, red blood cell disorders, inborn errors of metabolism, and severe immune deficiencies.^1^ Its clinical efficacy relies critically on the engraftment of donor hematopoietic cells, which regenerate all blood lineages. Insufficient hematopoietic regeneration, known as poor graft function (PGF) is a clinical condition, characterized by persistent cytopenias despite full donor chimerism.^2^, ^3^, ^4^ PGF is distinct from graft rejection, in which donor chimerism is lost due to the destruction of donor hematopoietic cells by residual immune‐effector cells of the recipient.^5^ Due to the lack of differentiated blood cells, HCT recipients with PGF are prone to infections and bleeding complications and have increased overall mortality.^2^ Consequently, a timely and accurate diagnosis of PGF is imperative to enable adequate treatment.

Currently, the assessment of PGF relies on the evaluation of post‐transplant blood counts (thrombocytes, neutrophils, and hemoglobin) relative to predefined cytopenic thresholds. After considering certain inclusion and exclusion criteria, these multiparameter blood count evaluations are condensed into a single, bimodal diagnosis of PGF or good graft function (GGF). However, numerous definitions for PGF exist. In a recent meta‐analysis,^6^ we identified 63 different definitions among 69 studies. Some studies consider PGF as a subtype of graft failure, requiring severe cytopenias,^7^, ^8^ while, in other studies, mild cytopenias are sufficient to diagnose PGF.^9^, ^10^ Three definitions for PGF were used most commonly, with an additional 60 definitions likely derived from the three core definitions^10^, ^11^, ^12^ (Table 1). The impact of this heterogeneity on the reported incidence, risk factors, and outcome of PGF remains unknown. This knowledge gap has important consequences, limiting the cross‐comparability of different observational studies or transplant registries and hindering the development and evaluation of preventive and treatment strategies for PGF. In addition, the lack of a standardized, quantitative definition of PGF complicates both clinical decision‐making for individual patients and broader epidemiologic or fundamental research efforts.

**Table 1:** PGF criteria per definition.

Criterion	Kong et al. (2013)	Klyuchnikov et al. (2014)	Stasia et al. (2014)
Number of cytopenic lineages required for PGF	2 or 3	2 or 3	2 or 3
Threshold for thrombocytopenia	20 × 10^9^/L	30 × 10^9^/L	30 × 10^9^/L
Threshold for neutropenia	0.5 × 10^9^/L	1.5 × 10^9^/L	1.0 × 10^9^/L
Threshold for anemia	7 g/dL	10 g/dL	8.5 g/dL
Minimum duration of cytopenia	3 days	14 days	14 days
Time point after HCT from which blood counts are included for cytopenia	Day +28	Day +14	Day +14

Abbreviations: HCT, hematopoietic cell transplantation; PGF, poor graft function.

In recent years, advances in healthcare digitization have led to the availability of big data on hematopoietic reconstitution, as well as methods to analyze these data. Together, these may enable the development of new strategies to assess post‐transplant (poor) graft function and predict HCT outcomes. In this multicenter study, we used over 35.000 blood count measurements of 427 pediatric and 405 adult allo‐HCT recipients to classify patients as having PGF or GGF based on their hematological reconstitution. By manipulating the thresholds used to define PGF and the time after HCT at which these are applied, we assessed the consequences of existing heterogeneity in PGF definitions in these two separate cohorts. We reveal substantial disparities in PGF incidence, risk factors, and outcome, contingent on the specific PGF definition used. Our data underline the need for a standardized and quantitative definition to enable adequate identification and treatment of PGF.

## MATERIALS AND METHODS

### Patient cohorts

Pediatric and adult recipients of a first HCT were included in this study and analyzed as separate cohorts (Table 2). The pediatric cohort was transplanted between 2005 and 2019 in the Wilhelmina Children's Hospital (2005–2017) or the Princess Máxima Center (2017–2019). The latter center was opened in 2018, as a nationwide pediatric oncology referral center, and the pediatric HCT unit was moved from the Wilhelmina Children's Hospital to the Princess Máxima Center. The adult cohort was transplanted in the University Medical Center Utrecht (UMCU) between 2011 and 2019. All patients received post‐HCT follow‐up in their transplant centers.

**Table 2:** Characteristics of patient cohorts.

	Pediatric (*n* = 427)		Adult (*n* = 405)	
Determinant	*n*	%	*n*	%
Sex				
Male	241	(56.4)	247	(61.0)
Female	186	(43.4)	158	(39.0)
Age at transplant (years), median (IQR)	8.38	(2.4–13.4)	55.8	(41.2–63.2)
Underlying disease				
Hemato‐oncology	228	(53.4)	375	(92.6)
Bone marrow failure syndromes	72	(16.9)	17	(4.2)
Inherited disorders	60	(14.1)	8	(2.0)
Immunodeficiency	61	(14.3)	0	(0)
Other	6	(1.4)	5	(1.2)
Conditioning				
Chemotherapy‐based	382	(89.5)	372	(96.8)
TBI‐based	42	(10.5)	13	(3.2)
Serotherapy				
Yes	303	(71)	307	(75.8)
No	124	(29)	98	(24.2)
Stem cell source				
Cord blood	219	(51.3)	55	(13.6)
Bone marrow	184	(43.1)	24	(5.9)
Peripheral blood stem cells (PBSC)	5	(1.2)	322	(79.5)
Combination	19	(4.4)	4	(1.0)
Cord blood + PBSC	12		4	(1.0)
Double cord	6		0	(0.0)
Cord blood + Bone marrow	1		0	(0.0)
Donor age (years)^a^				
Including cord blood, median (IQR)	0	(0–22)	29.9	(22.4–45.1)
Excluding cord blood, median (IQR)	23.0	(13.0–31.0)	34.7	(25.6–47.7)
Missing	0	(0)	56	(13.8)
Relation to donor				
Unrelated	317	(74.2)	302	(74.6)
Related	91	(21.3)	99	(24.4)
Combination	19	(4.4)	4	(1.0)
HLA matching				
Full match	168	(39.3)	311	(76.8)
Mismatch	146	(34.2)	90	(22.2)
Missing	94	(22.0)	0	(0)
Combination	19	(4.4)	4	(4.4)

Abbreviations: HLA, human leukocyte antigen; IQR, interquartile range; TBI, total body irradiation.

a Excluding patients transplanted with combination grafts.

To enable the classification of patients into either PGF or GGF, patients who were deceased within 28 days after the transplant were excluded (Supporting Information S1: Figure S1). In addition, to allow meaningful longitudinal analysis of blood counts, patients were excluded if they had less than two blood measurements between Day 28 and Day 100 post‐HCT. This study was approved by local ethical committees (#11‐063k for the pediatric cohort and #24U‐1439 for the adult cohort), and all patients provided written informed consent for the use of their data retrieved around HCT in accordance with the Declaration of Helsinki.

### HCT procedures

Patients were transplanted in high‐efficiency, particle‐free, air‐filtered, positive‐pressure isolation rooms. Conditioning regimens followed (inter)national disease‐specific guidelines.^13^, ^14^, ^15^ Prior to 2018, the majority of adult patients received a myeloablative conditioning (MAC; age ≤ 40 years) or reduced intensity conditioning (RIC; age > 40 years), followed by a T cell replete mobilized peripheral blood stem cell (PBSC) allograft. As of 2018, the majority of adult patients received MAC (regardless of age, followed by an ex vivo T cell‐depleted allo‐HCT as previously described.^16^, ^17^ Patients underwent selective gut decontamination and received infection prophylaxis. Thymoglobulin was mostly used as serotherapy. For pediatric patients, GvHD prophylaxis consisted of cyclosporin A (CsA) combined with either prednisolone (cord blood graft recipients) or methotrexate (bone marrow graft recipients). For adult patients, GvHD prophylaxis consisted of CsA and mycophenolate mofetil (MMF) (T cell replete graft recipients) or MMF only (ex vivo T cell‐depleted graft recipients).

### Definitions

Patients were classified as having PGF at any time based on the three most commonly used, distinct definitions used in literature,^10^, ^11^, ^12^ as demonstrated in our previous meta‐analysis.^6^ In each of these definitions, PGF is defined by the presence of cytopenia in two or three lineages (i.e., hemoglobin, neutrophils, thrombocytes) simultaneously. The specific cytopenic thresholds, duration criteria, and the time point after HCT at which cytopenias were considered significant are shown in Table 1. Neutrophil engraftment was defined as neutrophils above 0.5 × 10^9^/L for 3 consecutive days after Day 10. Thrombocyte engraftment was defined as thrombocytes above 20 × 10^9^/L for 3 consecutive days after Day 10, excluding measurements on or 1 day surrounding the administration of thrombocyte transfusions. PGF was considered primary if no neutrophil engraftment occurred prior to the onset of PGF, and secondary if neutrophil engraftment was achieved before PGF onset. PGF was considered bilineage if two cytopenias were present simultaneously in the entire follow‐up period, and trilineage if all three lineages were cytopenic simultaneously. Graft rejection was based on clinical diagnosis and extracted from clinical charts.

### Identification of patients with PGF

Post‐HCT blood counts were extracted from the Utrecht Patient Oriented Database (UPOD)^18^ and used to computationally screen each individual for the presence of PGF, using the three most commonly used definitions described above. UPOD is a system of relational databases that captures routine care data for all patients who visit the UMCU/Princess Máxima Center. Specifically, within UPOD, for all patients undergoing any type of routine hematology analysis in either red or white blood cells or platelets (such as hemoglobin, white blood cell count, or platelet count), a complete blood count including research‐only values and raw blood cell characteristics is stored. This means all counts are available for all patients. Only data from the first HCTs were included. Data were analyzed from the day of HCT (index date) until 2 years post‐HCT. For patients undergoing a second HCT, blood counts from 10 days prior to the second transplant onwards were excluded to eliminate effects from the associated conditioning regimen. In cases of multiple measurements on a single day, the lowest value for each lineage was used. Per lineage, all days below the cytopenic threshold were identified. Subsequently, consecutive days below the threshold were counted if they fulfilled the duration requirement of each specific PGF definition (Table 1). Patients were classified as having PGF if cytopenias were present in two or three lineages simultaneously.

### Computations and statistical methods

The data preparation and statistical analyses were performed in RStudio 2022.02.1^19^ using R version 4.3.1.^20^ LOESS‐regression curves were used to visualize blood count data. Cumulative incidence analyses were conducted using the {tidycmprsk} package, correcting for competing risks. In line with existing definitions of PGF,^10^, ^11^, ^12^ post‐HCT relapse, severe (grade III–IV) acute graft‐versus‐host‐disease (aGvHD), and/or graft rejection were treated as competing risks for PGF. Cytopenias occurring after or within 3 days prior to the onset of these complications were not classified as PGF.

Mortality was assessed within the first 2 years post‐HCT by cumulative incidence of death. To this end, patients were retrospectively classified into PGF or GGF, based on the development of PGF within the first 2 years post‐HCT. For mortality, no competing risks were included. For the cumulative incidence of stem cell boost and/or retransplantation, relapse and death of any cause were included as competing risks.

Risk factors were evaluated in univariable cause‐specific hazard models of PGF. In the case of missing donor age, median imputation was performed, matching for stem cell source and donor relation.

## RESULTS

### Patient characteristics

Between 2005 and 2019, 451 pediatric (2005–2019) and 435 adult patients (2011–2019) received their first HCT in the Princess Máxima Center/Wilhelmina Children's Hospital or UMC Utrecht. From these HCT recipients, 24 pediatric and 30 adult patients were excluded because of death within the first 28 days after HCT (6 pediatric and 7 adult patients) or because of insufficient blood count data (18 pediatric and 23 adult patients), which prevented reliable assessment of (poor) graft function. In total, 427 pediatric and 405 adult HCT recipients were included in our analyses (Supporting Information S1: Figure S1).

Clinical characteristics are summarized in Table 2. The median age at transplant was 8.4 years (interquartile range [IQR]: 2.4–13.4 years) for pediatric recipients and 55.8 (IQR: 41.2–63.2) for adults. The most common indication for HCT was hemato‐oncology (53.4% and 92.6%, respectively). Stem cell sources were cord blood (51.3% and 13.6%), bone marrow (43.1% and 5.9%), peripheral blood (1.2% and 79.5%), or a combination of multiple grafts (4.4% and 1.0%). Given the differences in clinical characteristics between the pediatric and adult cohorts, we subsequently analyzed these cohorts separately.

### Longitudinal assessment of hematopoietic reconstitution after HCT

**Figure 1:**
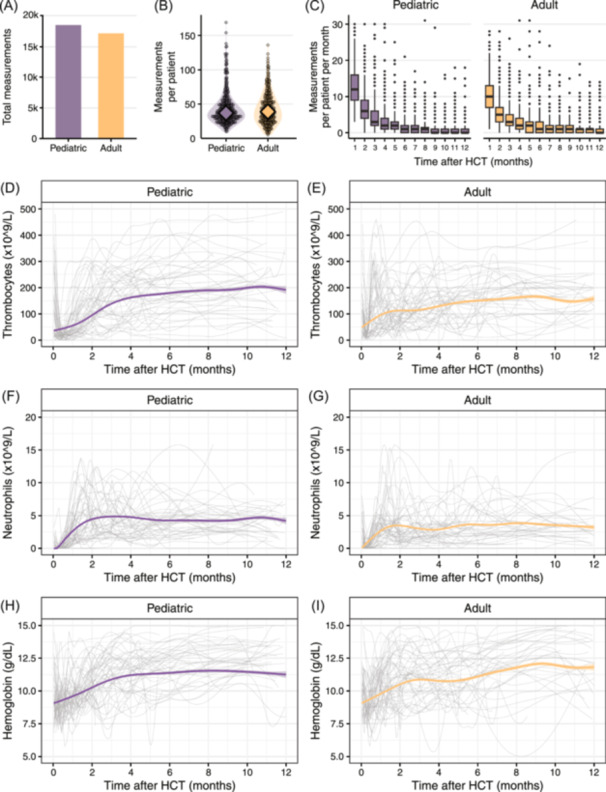
**Hematopoietic reconstitution after hematopoietic cell transplantation. (A)** Bar plots showing total measurements in the pediatric (purple) and adult cohort (amber) within 2 years after HCT. **(B)** Violin plots total measurements per patient. Diamonds depict median values. **(C)** Box plots showing the number of measurements per patient within a given month after HCT. **(D–I)** LOESS curves visualizing recovery of thrombocytes **(D, E)**, neutrophils **(F, G)**, and hemoglobin **(H, I)** in the first year after HCT. Purple and amber lines with shading show the local estimate with a 95% confidence interval for the total pediatric and adult cohorts, respectively. Gray lines show the LOESS curves for 50 individuals randomly sampled from each cohort. HCT, hematopoietic cell transplantation.

To evaluate post‐transplant hematopoietic reconstitution and engraftment kinetics, blood count measurements were extracted and visualized on an individual and cohort level (Figure 1). The median number of blood count measurements per patient within the first 2 years post‐HCT was 38 for pediatric patients and 39 for adults (range: 11–170 and 6–137, respectively, Figure 1B). Based on longitudinal blood count measurements, reconstitution dynamics could be analyzed (Figure 1D–I, Supporting Information S1: Figure S2). Neutrophil engraftment occurred at a median of 24 days (IQR: 19–29 days) after HCT in pediatric recipients and a median of 19 days (IQR: 15–25 days) in adults. Thrombocyte engraftment took a median of 21 days (IQR: 14–32 days) for children and 13 days (IQR: 11–19 days) for adults. Reconstitution dynamics varied by graft source (Supporting Information S1: Figure S2D–F). For instance, in adult recipients of cord blood grafts, both neutrophil and thrombocyte engraftment took longer compared to adult recipients of other stem cell sources. In pediatric HCT recipients, neutrophil engraftment was similar between bone marrow and cord blood recipients, which is likely due to the standard administration of granulocyte‐colony stimulating factor (G‐CSF) in pediatric cord blood recipients.

### Classification based on longitudinal blood counts reveals definition‐dependent differences in PGF incidence

Subsequently, we used these longitudinal blood counts to classify HCT recipients as having either PGF or GGF and assessed the consequences of differences in PGF definitions. To this end, we developed an analytical pipeline that uses thresholds for depth, timing, and duration of cytopenias to classify each patient as PGF or GGF, according to the specific definition being tested. We then applied this approach to identify PGF patients according to the three most frequently used definitions in literature (Table 1).

**Figure 2:**
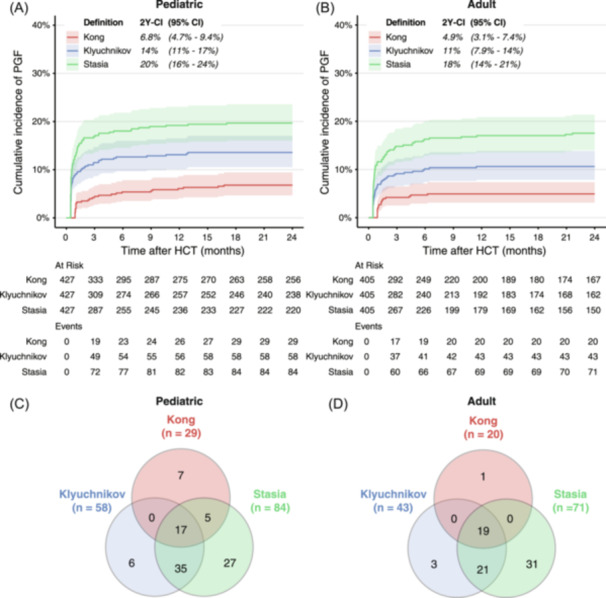
**Comparison of PGF incidence according to different definitions. (A, B)** Cumulative incidence of PGF according to the three most common definitions used in literature, in pediatric **(A)** and adult **(B)** HCT recipients, with graft rejection, retransplantation, and death prior to PGF as competing risks. Shaded areas depict a 95% confidence interval. For each definition, the cumulative incidence at two years after HCT (2Y‐CI), including a 95% confidence interval (95% CI), is shown at the top of the panel. **(C, D)** Venn diagrams depicting the number of PGF patients defined by one, two, or all three definitions for the pediatric **(C)** and adult cohort **(D)**. 2Y‐CI, two‐year cumulative incidence; CI, confidence interval; HCT, hematopoietic cell transplantation; PGF, poor graft function.

Interestingly, both in pediatric and adult HCT recipients, the number of recipients classified as PGF varied significantly, depending on the chosen definition (Figure 2). For instance, in the pediatric cohort, the 2‐year cumulative incidence of PGF was 6.8% (95% confidence interval [CI]: 4.7%–9.4%) according to the most stringent definition (“Kong”), compared to 14% or 20% when less stringent definitions were used. Among the 97 pediatric patients classified as PGF by at least one definition, 17 patients (17.5%) were classified by all three definitions, 40 (41%) were classified by two definitions, and another 40 (41%) were classified as PGF by one definition only (Figure 2C). The adult cohort showed very similar results, with cumulative PGF incidence ranging from 4.9% for the most stringent (“Kong”) up to 18% for the most lenient definition (“Stasia,” Figure 2B). The distribution of PGF subtypes, including primary/secondary and bilineage/trilineage PGF, are shown in Table S1.

Since post‐HCT complications such as relapse and severe aGvHD may result in cytopenias, cytopenias and/or PGF may be present in patients shortly before they are diagnosed with these complications. When comparing different definitions, the percentage of PGF patients that developed severe aGvHD or relapse after PGF onset were similar (Supporting Information S1: Figure S3A), indicating that the definition‐dependent differences in PGF incidence were not due to differential inclusion of patients developing aGvHD and/or relapse. Sensitivity analysis, excluding patients who developed these complications within 60 days after PGF, produced similar definition‐dependent differences in PGF incidence (Supporting Information S1: Figure S3). Together, our findings show that the choice of definition significantly affects the number of patients classified as PGF, resulting in up to three‐fold differences among the tested definitions in both pediatric and adult HCT recipients.

### Stringent PGF definitions identify HCT recipients with high mortality

**Figure 3:**
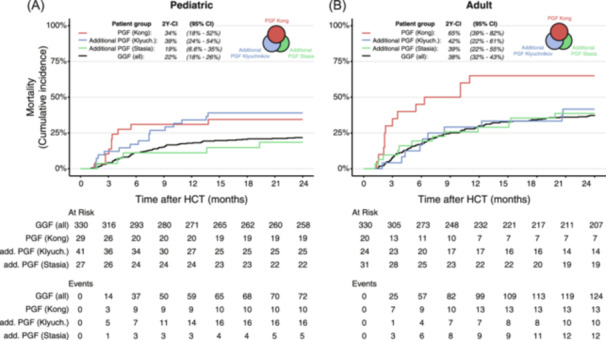
**Comparison of mortality in different subsets of PGF and GGF patients. (A, B)** Cumulative incidence of mortality after HCT in patients with PGF or GGF in the pediatric **(A)** and adult cohorts **(B)**. The total number of PGF patients is split into those classified by Kong, the additional patients that are classified by Klyuchnikov (but not Kong), and the additional patients classified by Stasia (but not Kong or Klyuchnikov), as depicted in the inlay in the top right of each panel. For each subgroup, the cumulative incidence of mortality at 2 years after HCT (2Y‐CI) including a 95% confidence interval (95% CI) is shown at the top of the panel. 2Y‐CI, two‐year cumulative incidence; add., additional; CI, confidence interval; GGF, good graft function; HCT, hematopoietic cell transplantation; Klyuch., Klyuchnikov; PGF, poor graft function.

Next, we investigated whether PGF patients also showed definition‐dependent differences in post‐HCT outcome. For all definitions, 2‐year post‐HCT mortality was higher for PGF patients compared to HCT recipients with GGF (Supporting Information S1: Figure S4), although these differences were not significant. In the adult cohort, mortality was highest for the most stringent definition (“Kong”), requiring severe cytopenias (mortality: 65%, 95% CI: 39%–82%, Figure 3B), and lowest for the definition including mild cytopenias (“Stasia,” mortality: 46%, 95% CI: 34%–57%). In the pediatric cohort, mortality was highest for patients classified by Klyuchnikov (40%), followed by Kong (34%) and Stasia (33%). This is surprising since one might expect that patients with the most severe cytopenias would have the worst outcome. Notably, differences in PGF outcome may have been obscured by an increased tendency for clinicians to recognize and treat more severe PGF. In line with this thought, the 2‐year cumulative incidence of retransplantation or stem cell boost in pediatric PGF patients classified by Kong was 45% (95% CI: 26%–62%), compared to 21% (95% CI: 11%–32%) for Klyuchnikov and 19% for Stasia (95% CI: 11%–28%, Supporting Information S1: Figure S5).

Given our finding that different PGF definitions identify both overlapping and non‐overlapping PGF patients (Figure 2C,D), PGF survival may be to a large degree determined by the overlapping patients. To investigate if the use of lenient PGF definitions resulted in the additional classification of PGF patients with lower mortality, we next compared patients in four subgroups: (1) those classified by Kong; (2) additional PGF patients, classified by Klyuchnikov but not by Kong; (3) additional PGF patients classified by Stasia, but not by Kong or Klyuchnikov; and (4) HCT recipients with GGF according to all three definitions (Figure 3). In the adult cohort, patients with severe PGF, identified by Kong only, showed a mortality of 65% (95% CI: 39%–82%). Contrastingly, the mortality of the additional PGF patients identified by Klyuchnikov (42%, 95% CI: 22%–61%) and Stasia (39%, 95% CI: 22%–55%) was very similar to the mortality of HCT recipients with GGF (38%, 95% CI: 32%–43%). In the pediatric cohort, patients identified by Kong, as well as the additional patients classified by Klyuchnikov had high mortality (34% and 39%, respectively), while the additional PGF patients identified by Stasia showed similar survival to HCT recipients with GGF (19% and 22%, respectively). The cumulative incidence of retransplantation and stem cell boost was also similar for the latter two groups (Supporting Information S1: Figure S6). Together, our data show that HCT recipients with PGF have poor outcomes, especially when stringent PGF definitions are used. While lenient PGF definitions may classify more patients as having PGF, the outcome of these additional patients may not be different from those with GGF, suggesting that more stringent definitions are preferred.

### Risk factors for PGF are dependent on the definition used

Finally, we investigated whether the choice of PGF definition also affects which risk factors for PGF are identified. To this end, we assessed the association between clinical characteristics and the incidence of PGF in univariable cause‐specific hazard models (Table 3). These analyses revealed definition‐dependent differences in which risk factors were significantly associated with PGF. For example, in adults, older recipient age was only identified as a significant risk factor for PGF defined by Klyuchnikov and Stasia, showing a similar but non‐significant effect for PGF defined by Kong. In adults, cord blood transplantation was identified as a risk factor by all three definitions. Of note, even if significance was not reached, the direction of effect was similar between definitions, suggesting that the difference in identified risk factors may, at least partially be explained by the number of PGF patients classified by each definition. Together, these data show that the choice of definition affects which clinical characteristics are identified as risk factors for PGF.

**Table 3:** Risk factor analysis for PGF.

	Pediatric			Adult		
Predictor	HR	95% CI	*p* Value	HR	95% CI	*p* Value
Year of HCT (per year increase)						
Kong et al.	1.02	0.93–1.11	0.68	0.93	0.77–1.11	0.41
Klyuchnikov et al.	1.04	0.98–1.10	0.25	0.91	0.81–1.02	0.12
Stasia et al.	1.01	0.97–1.07	0.51	0.92	0.84–1.01	0.08
Recipient sex (male vs. female)						
Kong et al.	1.23	0.58–2.61	0.58	1.53	0.59–3.98	0.39
Klyuchnikov et al.	1.47	0.86–2.53	0.16	1.10	0.59–2.03	0.77
Stasia et al.	1.51	0.96–2.36	0.07	1.01	0.63–1.64	0.94
Recipient age (per year increase)						
Kong et al.	1.04	0.98–1.11	0.20	1.03	1.00–1.07	0.06
Klyuchnikov et al.	**1.05**	**1.00–1.19**	**0.04**	1.01	0.99–1.03	0.42
Stasia et al.	**1.04**	**1.00–1.08**	**0.03**	1.00	0.99–1.02	0.59
Underlying disease (Other vs. HemOnc)						
Kong et al.	0.64	0.30–1.37	0.25	*NE* a	*NE* a	*NE* a
Klyuchnikov et al.	**0.44**	**0.25–0.77**	**0.004**	1.28	0.46–3.60	0.63
Stasia et al.	**0.58**	**0.37–0.90**	**0.02**	1.17	0.51–2.70	0.71
Conditioning (TBI vs. chemotherapy)						
Kong et al.	0.30	0.04–2.19	0.23	**2.79**	**1.14–6.82**	**0.02**
Klyuchnikov et al.	0.77	0.31–1.92	0.57	**2.4**	**1.34–4.47**	**0.004**
Stasia et al.	0.88	0.43–1.83	0.74	**2.47**	**1.55–3.94**	**<0.001**
Serotherapy (yes vs. no)						
Kong et al.	0.87	0.39–1.90	0.72	0.42	0.17–1.03	0.06
Klyuchnikov et al.	0.62	0.37–1.06	0.08	**0.46**	**0.25–0.86**	**0.01**
Stasia et al.	1.04	0.64–1.68	0.87	**0.41**	**0.25–0.67**	**<0.001**
Stem cell source (CB vs. BM)^b^						
Kong et al.	1.25	0.58–2.69	0.57	*NE* a	*NE* a	*NE* a
Klyuchnikov et al.	1.10	0.64–1.89	0.73	1.84	9.68–5.04	0.23
Stasia et al.	1.28	0.81–2.01	0.29	**2.66**	**1.16–6.13**	**0.02**
Stem cell source (PBSC vs. BM)^b^						
Kong et al.	*NE* a	*NE* a	*NE* a	*NE* a	*NE* a	*NE* a
Klyuchnikov et al.	1.77	0.24–13.1	0.57	**0.31**	**0.12–0.83**	**0.02**
Stasia et al.	2.68	0.64–11.2	0.18	**0.36**	**0.16–0.81**	**0.01**
Stem cell source (CB vs. PBSC)^a^						
Kong et al.	*NE* a	*NE* a	*NE* a	**5.14**	**2.09–12.6**	**<0.001**
Klyuchnikov et al.	0.62	0.08–4.55	0.64	**5.85**	**3.07–11.2**	**<0.001**
Stasia et al.	0.48	0.12–1.96	0.31	**2.76**	**1.23–6.19**	**<0.001**
Donor relation (unrelated vs. family)^a^						
Kong et al.	2.37	0.71–7.87	0.16	6.68	0.89–49.9	0.06
Klyuchnikov et al.	1.67	0.79–3.54	0.18	1.61	0.84–3.06	0.15
Stasia et al.	**2.06**	**1.06–4.00**	**0.03**	**2.34**	**1.28–4.27**	**0.01**
Donor sex (male vs. female)^b^						
Kong et al.	0.78	0.36–1.65	0.51	2.53	0.84–7.63	0.10
Klyuchnikov et al.	1.01	0.59–1.75	0.95	1.69	0.86–3.29	0.13
Stasia et al.	0.95	0.61–1.49	0.84	1.47	0.89–2.43	0.14
Donor age (per year increase)^b^,^c^						
Kong et al.	1.04	0.99–1.09	0.11	0.99	0.95–1.04	0.79
Klyuchnikov et al.	1.02	0.99–1.06	0.17	1.01	0.99–1.05	0.27
Stasia et al.	1.03	1.00–1.06	0.08	1.01	0.98–1.03	0.53
HLA matching (mismatch vs. full match)^b^						
Kong et al.	1.38	0.58–3.25	0.47	**5.95**	**2.45–14.6**	**<0.001**
Klyuchnikov et al.	**2.19**	**1.14–4.21**	**0.02**	**4.33**	**2.38–7.88**	**<0.001**
Stasia et al.	**2.70**	**1.54–4.74**	**<0.001**	**3.05**	**1.90–4.90**	**<0.001**

*Note:* Results with p < 0.05 are shown in bold.

Abbreviations: BM, bone marrow; CB, cord blood; CI, confidence interval; HemOnc, hemato‐oncology; HR, hazard ratio; PBSC, peripheral blood stem cells; TBI, total body irradiation.

^a^ Non‐estimable (NE) due to zero patients with PGF in the subgroup

^b^ Excluding patients transplanted with combination grafts (i.e., combination from two separate donors).

^c^ Corrected for stem cell source.

## DISCUSSION

Over the past decades, PGF has been increasingly recognized as a severe yet common complication of HCT.^2^ However, how to define PGF remains unclear, with variability between published studies^6^ and international guidelines.^3^, ^21^ Here, using longitudinal analyses of over 35.000 blood count values, we systematically analyze the impact of existing heterogeneity in PGF definitions on its reported incidence, risk factors, and outcome. We show that the number of patients classified as PGF differed more than threefold, depending on the definition used. Stringent PGF definitions identify HCT recipients with high mortality, while the additional patients classified by lenient definitions resemble patients with GGF. Lastly, identified risk factors differ contingent on the definition.

The absence of a quantitative, uniform definition of PGF hampers clinical decision‐making at the level of individual patients, as well as epidemiologic and fundamental investigations into PGF. Current PGF definitions differ in various aspects, including differences in cutoff values used to define cytopenias. In this work, we demonstrate how changes in these cutoff values affect the reported incidence, identified risk factors, and outcome of PGF, highlighting the need for a standardized PGF definition, including requirements for cytopenia severity. The need for a well‐defined, uniform definition has been recognized previously by us^6^ and others,^4^, ^21^ and recently, both the European Society for Blood and Marrow Transplantation (EBMT) and the American Society for Transplantation and Cellular Therapy (ASTCT) have published their consensus definitions for PGF.^3^, ^21^ While these definitions are an important step toward harmonization, it should be noted that neither definition provides quantitative cutoffs to define PGF, leaving room for heterogeneity. A uniform, quantitative definition is required to improve our understanding of PGF pathophysiology and enable investigations into appropriate treatment strategies, as described below.

First, a standardized and quantitative PGF definition may enable the identification of PGF subtypes with different pathophysiology. The pathophysiology of PGF is likely multifactorial, including factors related to the quality^22^ and quantity^8^, ^23^, ^24^ of donor stem cells, factors influencing the condition of the recipient bone marrow niche (including recipient age,^25^ underlying disease,^9^, ^11^ and previous chemotherapy^26^, ^27^), and post‐transplant events resulting in damage to or inhibition of hematopoietic function (including infections,^24^, ^25^ inflammation,^28^, ^29^ or drug‐related myelosuppression^30^, ^31^). These different pathophysiological processes might dictate the timing, duration, or severity of cytopenias. For example, niche‐related PGF could manifest as mild cytopenias, while stem cell‐related PGF might result in more severe cytopenias. In line with this hypothesis, we show that the identified risk factors are contingent on the PGF definition. Harmonization of PGF definitions will enable meaningful comparison of studies and identification of shared risk factors, which may reflect key processes in the pathophysiology of PGF. In addition, by focusing on PGF with either severe or mild cytopenias only, potential differences in underlying pathology may be identified.

Second, a standardized and quantitative PGF definition is required to optimize treatment and improve the outcome of PGF patients. Based on our mortality data, patients with PGF identified only by Stasia have milder PGF compared to those also identified by other definitions. The relative outcome of additional PGF patients identified by Klyuchnikov, but not by Kong, differed between cohorts. In the pediatric cohort, these patients showed high mortality, while in adults, mortality was similar to recipients with GGF. While mild PGF may be transient and adequately treated with supportive therapy, more severe PGF may require additional treatment. Differentiating between mild and severe PGF, and between transient and persistent PGF is crucial to identify optimal treatment strategies. Notably, current treatment options for PGF are limited. While stem cell boost or retransplantation can be used to treat PGF, these therapies are restricted by patient condition and donor cell availability. Alternatively, medications such as G‐SCF or thrombopoietin may be appropriate in certain PGF patients and are more readily available. Unfortunately, data on these medications were not available in our study. A harmonized PGF definition that distinguishes mild and severe PGF based on cytopenia severity will enable the investigation of these subtypes separately. Consequently, potential risks and benefits of available treatment options can be balanced appropriately, optimizing treatment for each type of PGF.

To our knowledge, this is the first study to directly compare the reported incidence, risk factors, and outcome of PGF across a range of definitions. By applying different definitions within the same cohort, we were able to specifically investigate definition‐dependent effects. In addition, by performing a side‐by‐side comparison of two separate cohorts, with different compositions in terms of age, underlying disease, and stem cell source, we demonstrate that definition‐dependent differences are present, both in pediatric HCT recipients and in adults. Several limitations should also be noted. First, in order to strictly adhere to PGF definitions, patients who were deceased prior to Day 28 or were retransplanted prior to Day 38 could not be classified as either PGF or GGF and were excluded from our analysis. Importantly, these patients represented less than 3% of both cohorts and were therefore unlikely to affect our findings. Second, given the retrospective nature of our study, treatment and outcome measures should be interpreted with care, since treatment decisions might have been affected by clinical parameters, including cytopenic depth, thereby selectively improving outcomes for specific patient subgroups. Finally, we limited our analysis to univariable models. These analyses already showed that different definitions affect the identified risk factors. More complex multivariable analyses were avoided, due to the small number of PGF cases, particularly for PGF defined by Kong, and the associated risk of overfitting.

Together, our findings demonstrate that differences in PGF definitions significantly affect its reported incidence, identified risk factors, and outcome. Based on our data, PGF subtypes with distinct outcomes may exist, which are represented differently between current definitions. According to current practice, both mild and severe PGF may benefit from treatment in the form of intensified supportive care, G‐CSF, or thrombopoietin agonists. Our data indicate that patients with mild PGF have similar mortality compared to HCT recipients with GGF, suggesting that high‐risk treatment options, such as stem cell boost or retransplantation, should potentially be reserved for patients with PGF according to stringent definitions only. To understand which treatment is optimal for each PGF subtype, future studies comparing these entities are required. We urge for a standardized definition, including quantitative thresholds for the depth, timing, and duration of cytopenias. This will homogenize PGF research, enabling meaningful interpretation of PGF incidence and outcomes across studies and over time facilitating translation from study findings into treatment decisions. A definition that differentiates mild and severe PGF, based on cytopenic severity, may enable a better understanding of PGF pathology and identification of optimal treatment strategies for all patients with PGF.

## AUTHOR CONTRIBUTIONS

*Conceptualization*: Konradin F. Müskens and Mirjam E. Belderbos. *Data analysis*: Konradin F. Müskens, Winny N. R. Collot‐d'Escury, and Rana Dandis. *Writing (original draft)*: Konradin F. Müskens and Mirjam E. Belderbos. *Supervision*: Mirjam E. Belderbos, Caroline A. Lindemans, Stefan Nierkens. *Funding acquisition*: Mirjam E. Belderbos. *Writing (review and editing)*: All authors. All authors have read and agreed to the published version of the manuscript.

## CONFLICT OF INTEREST STATEMENT

J. K. received grants from Novartis, Miltenyi Biotech, and Gadeta. He is a shareholder of Adeta and Ventury Therapeutics via Gadeta Founders and an inventor on multiple patents dealing with gdTCR and the development of engineered immune cells. C. A. L. is on a data and safety monitoring board for ExCellThera and was a medical advisor for Sobi and Orchard Therapeutics. S. N. was an advisor for Sobi. The remaining authors declare no conflicts of interest.

### FUNDING

This study was financially supported by a VENI grant of the Netherlands Organization for Scientific Research (NWO) (grant number VI.Veni.202.021 to M. E. B.), a physician‐scientist grant of the European Hematology Association (to M. E. B.), a John Hansen research grant from the DKMS, and a KiKa project grant (project number 418). The KiKa project grant supported the salary of KFM. The funders had no role in the design, execution, or writing of this work, nor in the decision to submit results.

## Supporting information

Supporting information.

Supporting information.

## Data Availability

The data that support the findings of this study are available on request from the corresponding author. The data are not publicly available due to privacy or ethical restrictions.
