# Supplementary material for: Definitions matter: Multicenter investigation of incidence and outcome of poor graft function after hematopoietic cell transplantation
Source: Hemasphere. 2024 Dec 17;8(12):e70059. doi: 10.1002/hem3.70059 (PMC11650888; doi:10.1002/hem3.70059)
Supplement: Supplementary file 1 — Supporting information. [file HEM3-8-e70059-s002.pdf]

Supplementary Figure 1

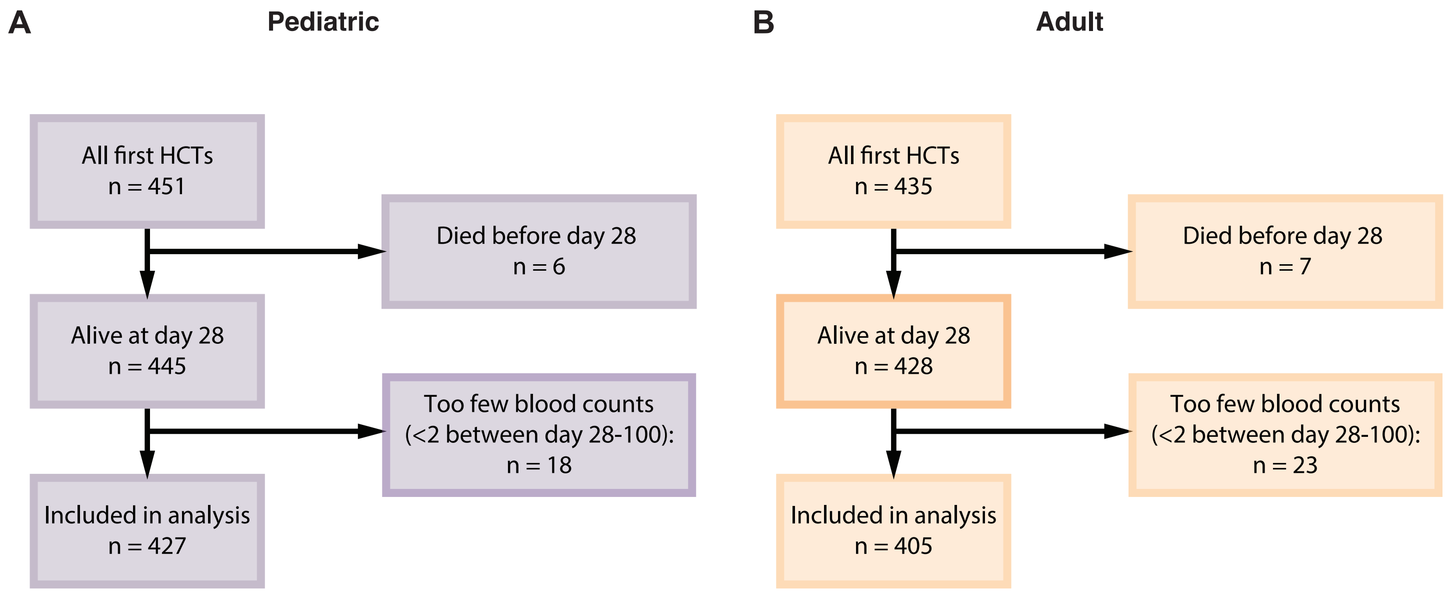

Supplementary Figure S1. Patient inclusion and exclusion. A-B) Venn diagrams showing the number of included and excluded patients within the pediatric (A) and adult cohort (B). Abbreviations: HCT: hematopoietic cell transplantation.

Supplementary Figure 2

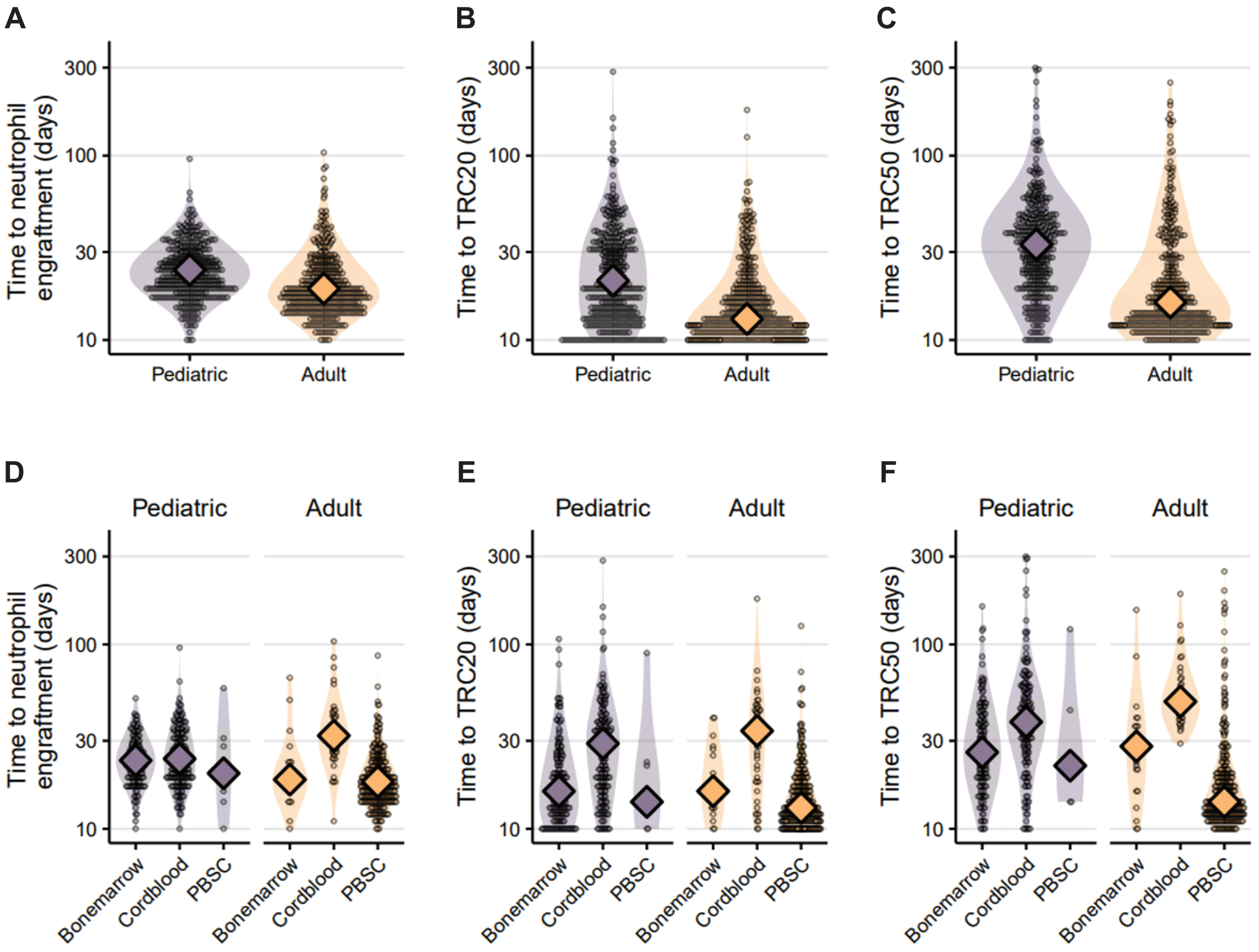

Supplementary Figure S2. Hematopoietic reconstitution dynamics. A-F) Violin plots depicting the time until neutrophil and thrombocyte engraftment, split by cohort, for all stem cell sources combined (A-C), or split per stem cell source (D-F). For thrombocyte engraftment, time until thrombocytes engraftment above 20 x 10^9 (TRC20) and 50 x 10^9 (TRC50) are shown. Abbreviations: PBSC: peripheral blood stem cells

Supplementary Figure 3

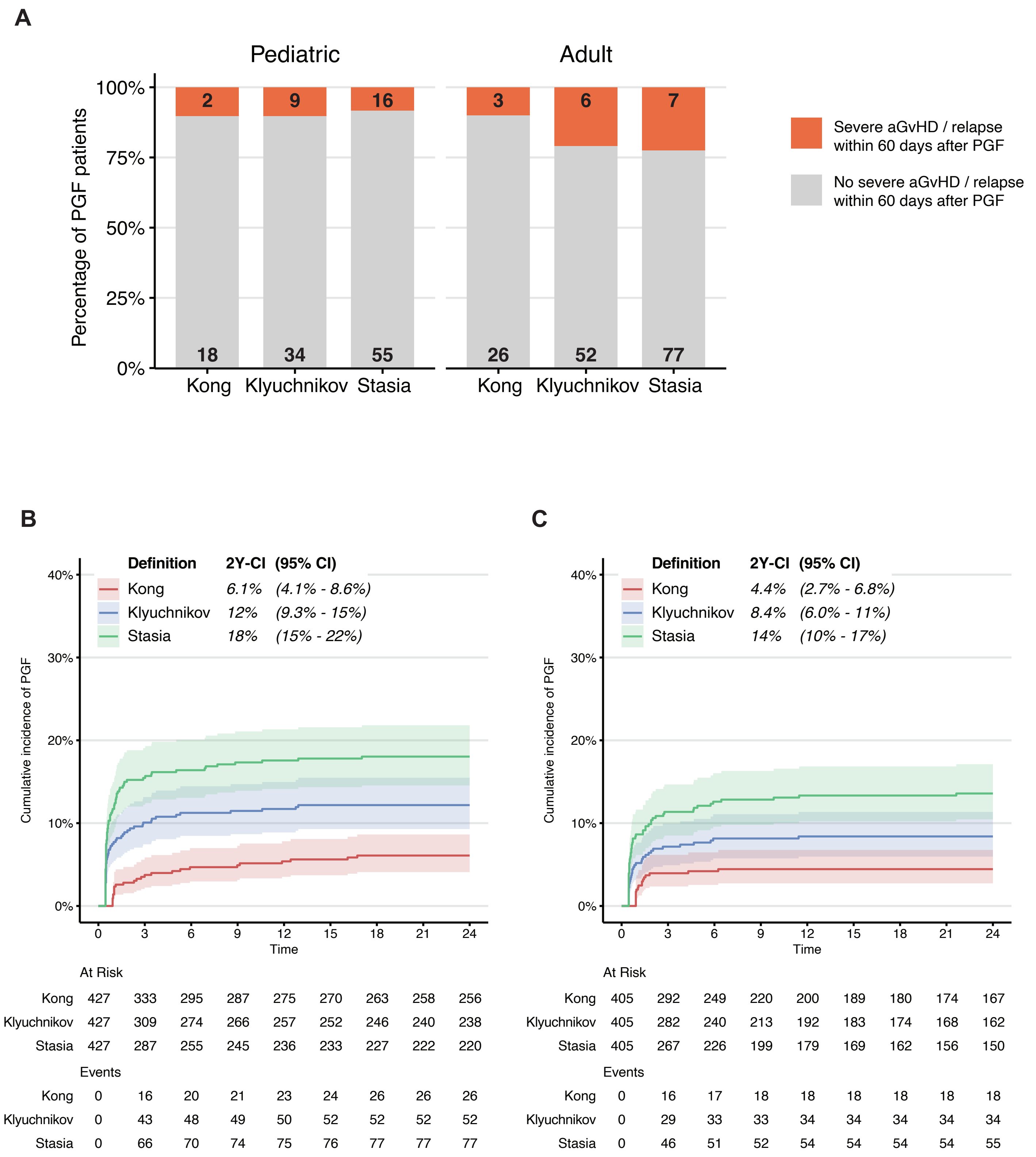

Supplementary Figure S3. Sensitivity analysis for PGF patients developing severe aGvHD or relapse. A) Bar plots depicting the percentage of PGF patients that developed severe acute graft-versus-host-disease (aGvHD) within 60 days after PGF onset for each definition, split into the pediaric (left panel) or adult cohort (right panel). Numbers within bars represent the total number of patients for each category. **B-C)** Cumulative incidence of PGF according to the three most common definitions used in literature, in pediatric (A) and adult (B) HCT recipients, excluding PGF patients who develop severe aGvHD or relapse within 60 days after PGF onset. Shaded areas depict 95% confidence interval. For each definition, the cumulative incidence at two years after HCT (2Y-CI) including 95% confidence interval (95% CI) are shown at the top of the panel. Abbreviations: PGF: poor graft function; aGvHD: acute graft-versus-host-disease.

Supplementary Figure 4

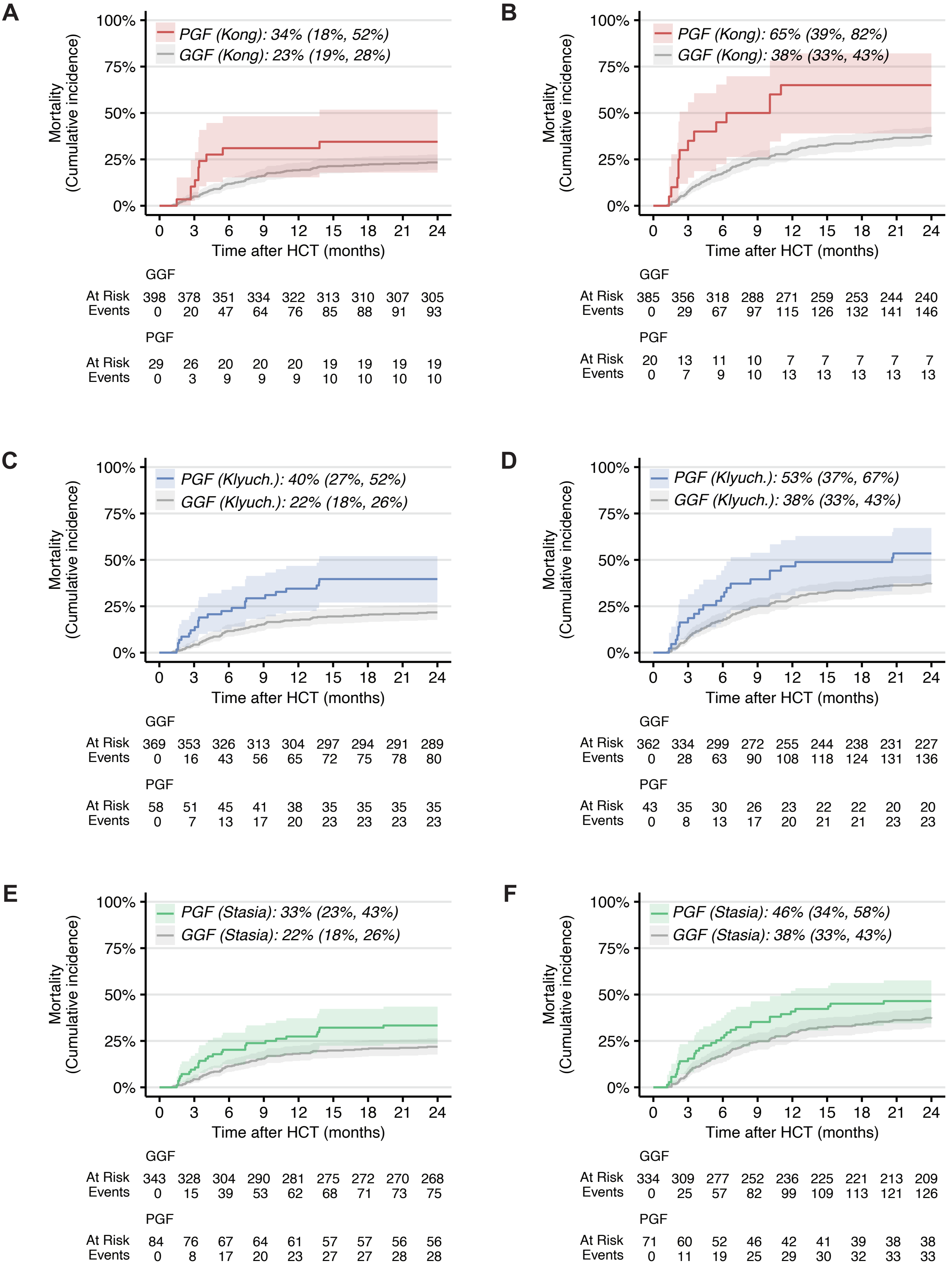

A

	0	3	6	9	12	15	18	21	24
GGF									
At Risk	398	378	351	334	322	313	310	307	305
Events	0	20	47	64	76	85	88	91	93
PGF									
At Risk	29	26	20	20	20	19	19	19	19
Events	0	3	9	9	9	10	10	10	10

B

	0	3	6	9	12	15	18	21	24
GGF									
At Risk	385	356	318	288	271	259	253	244	240
Events	0	29	67	97	115	126	132	141	146
PGF									
At Risk	20	13	11	10	7	7	7	7	7
Events	0	7	9	10	13	13	13	13	13

C

	0	3	6	9	12	15	18	21	24
GGF									
At Risk	369	353	326	313	304	297	294	291	289
Events	0	16	43	56	65	72	75	78	80
PGF									
At Risk	58	51	45	41	38	35	35	35	35
Events	0	7	13	17	20	23	23	23	23

D

	0	3	6	9	12	15	18	21	24
GGF									
At Risk	362	334	299	272	255	244	238	231	227
Events	0	28	63	90	108	118	124	131	136
PGF									
At Risk	43	35	30	26	23	22	22	20	20
Events	0	8	13	17	20	21	21	23	23

E

	0	3	6	9	12	15	18	21	24
GGF									
At Risk	343	328	304	290	281	275	272	270	268
Events	0	15	39	53	62	68	71	73	75
PGF									
At Risk	84	76	67	64	61	57	57	56	56
Events	0	8	17	20	23	27	27	28	28

F

	0	3	6	9	12	15	18	21	24
GGF									
At Risk	334	309	277	252	236	225	221	213	209
Events	0	25	57	82	99	109	113	121	126
PGF									
At Risk	71	60	52	46	42	41	39	38	38
Events	0	11	19	25	29	30	32	33	33

Supplementary Figure S4. Mortality of patients with PGF or GGF according to different definitions. A-F) Cumulative incidence of mortality after HCT in patients with PGF or GGF in the pediatric (A, C, E) and adult cohort (B, D, F). For each subgroup, the cumulative incidence of mortality at two years after HCT (2Y-CI) including 95% confidence interval (95% CI) are shown at the top of the panel. Abbreviations: 2Y-CI: Two-year cumulative incidence; CI: confidence interval; HCT: hematopoietic cell transplantation; GGF: good graft function; PGF: poor graft function, add.: additional.

Supplementary Figure 5

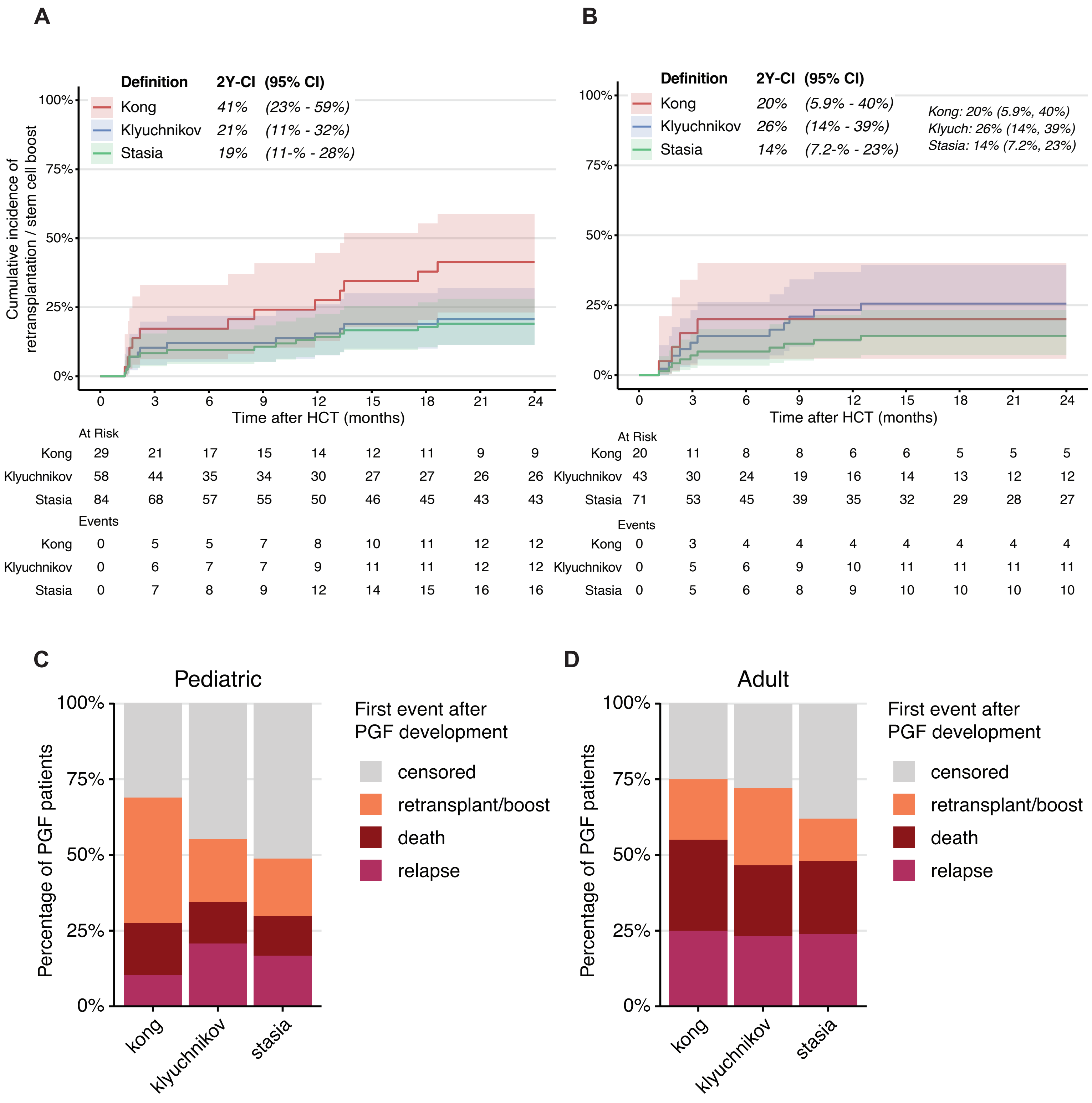

Supplementary Figure S5. Comparison of retransplantation and/or stem cell boost treatment in different subsets of PGF patients. A-B) Cumulative incidence of stem cell boost and/or retransplantation treatment in patients with PGF according to the three most common definitions used in literature, in pediatric (A) and adult (B) HCT recipients. Relapse and death prior to treatment were included as competing risks. Shaded areas depict 95% confidence interval. For each definition, the cumulative incidence of retransplantation/boost at two years after HCT (2Y-CI) including 95% confidence interval (95% CI) are shown at the top of the panel. **C-D)** Bar plots depicting the first event that occurred after PGF (defined by the definition shown), in the pediatric (C) and adult cohort (D). Abbreviations: 2Y-CI: Two-year cumulative incidence; CI: confidence interval; HCT: hematopoietic cell transplantation; PGF: poor graft function.

Supplementary Figure 6

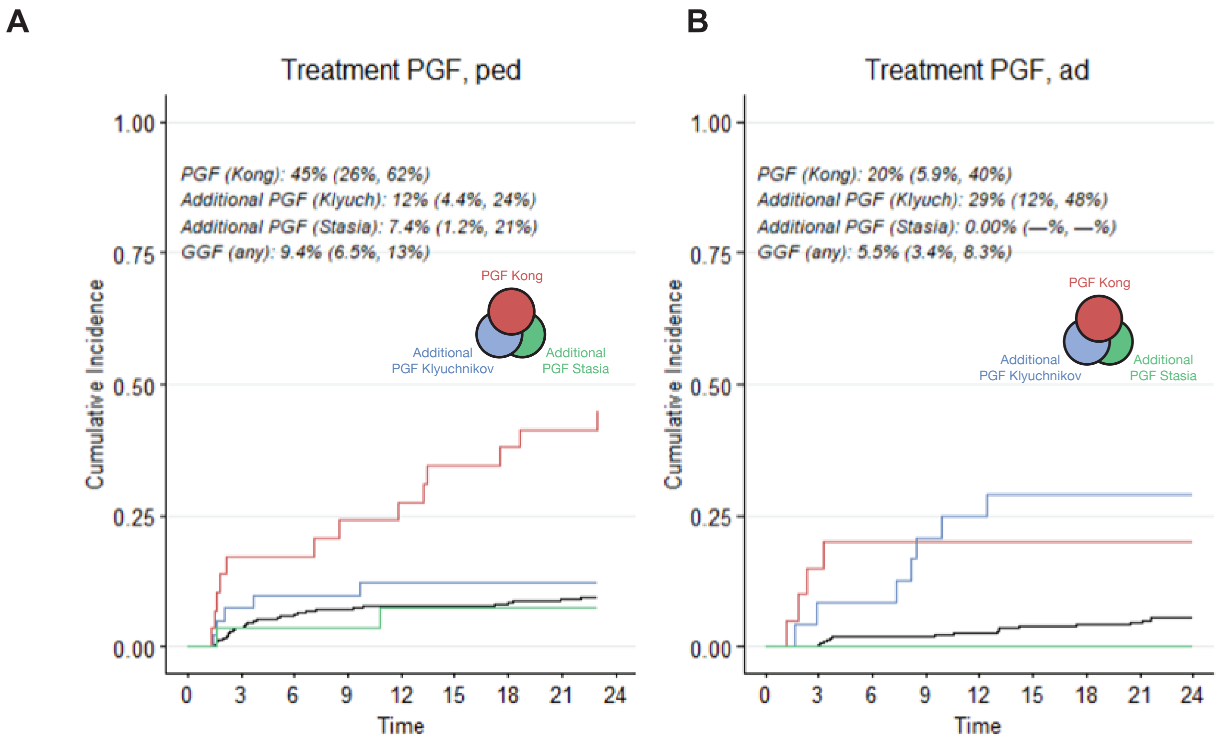

Supplementary Figure S6. Comparison of treatment in different subsets of PGF patients. A-B) Cumulative incidence of stem cell boost or retransplantation in patients with PGF or GGF in the pediatric (A) and adult cohort (B). The total number of PGF patients is split into those classified by Kong, the additional patients that are classified by Klyuchnikov (but not Kong), and the additional patients classified by Stasia (but not Kong or Klyuchnikov), as depicted in the inlay in top right of each panel. Death or relapse prior to treatment were included as competing risks. For each subgroup, the cumulative incidence of treatment at two years after HCT (2Y-CI) including 95% confidence interval (95% CI) are shown at the top of the panel. Abbreviations: 2Y-CI: Two-year cumulative incidence; CI: confidence interval; HCT: hematopoietic cell transplantation; GGF: good graft function; PGF: poor graft function, add.: additional.
