# Supplementary material for: Definitions matter: Multicenter investigation of incidence and outcome of poor graft function after hematopoietic cell transplantation
Source: Hemasphere. 2024 Dec 17;8(12):e70059. doi: 10.1002/hem3.70059 (PMC11650888; doi:10.1002/hem3.70059)
Supplement: Supplementary file 2 — Supporting information. [file HEM3-8-e70059-s001.docx]

**Supplementary table S1: PGF subtypes according to different definitions**

| **PGF subtype** | **Kong et al., 2013** | **Klyuchnikov et al., 2014** | **Stasia et al., 2014** |
| --- | --- | --- | --- |
|  |  |  |  |
| **Pediatric** | n = 29 | n = 58 | n = 84 |
|  |  |  |  |
| **Bilineage** | 27 (93%) | 54 (93%) | 64 (77%) |
| **Trilineage** | 2 (6.9%) | 4 (6.9%) | 20 (23%) |
|  |  |  |  |
| **Primary** | 13 (45%) | 38 (66%) | 51 (61%) |
| **Secondary** | 16 (55%) | 20 (34%) | 33 (39%) |
|  |  |  |  |
| **Adult** | n = 20 | n = 43 | n = 71 |
|  |  |  |  |
| **Bilineage** | 18 (90%) | 33 (77%) | 47 (66%) |
| **Trilineage** | 2 (10%) | 10 (23%) | 24 (34%) |
|  |  |  |  |
| **Primary** | 11 (55%) | 23 (53%) | 42 (59%) |
| **Secondary** | 9 (45%) | 20 (47%) | 29 (41%) |
|  |  |  |  |
